# *RMRP*, *RMST*, *FTX* and *IPW*: novel potential long non-coding RNAs in medullary thyroid cancer

**DOI:** 10.1186/s13023-020-01665-5

**Published:** 2021-01-06

**Authors:** Berta Luzón-Toro, Leticia Villalba-Benito, Raquel María Fernández, Ana Torroglosa, Guillermo Antiñolo, Salud Borrego

**Affiliations:** 1 Department of Maternofetal Medicine, Genetics and Reproduction, Institute of Biomedicine of Seville (IBIS), University Hospital Virgen del Rocío/CSIC, University of Seville, Seville, Spain; 2 Centre for Biomedical Network Research on Rare Diseases CIBERER, Seville, Spain

**Keywords:** lncRNA, MTC, MEN2, *RMST*, *FTX*, *IPW*, *RMRP*

## Abstract

The relevant role of long non-coding RNAs (lncRNAs) in cancer is currently a matter of increasing interest. Medullary thyroid cancer (MTC) is a rare neuroendocrine tumor (2–5% of all thyroid cancer) derived from the parafollicular C-cells which secrete calcitonin. About 75% of all medullary thyroid cancers are believed to be sporadic medullary thyroid cancer (sMTC), whereas the remaining 25% correspond to inherited cancer syndromes known as Multiple Endocrine Neoplasia type 2 (MEN2). MEN2 syndrome, with autosomal dominant inheritance is caused by germline gain of function mutations in *RET* proto-oncogene. To date no lncRNA has been associated to MEN2 syndrome and only two articles have been published relating long non-coding RNA (lncRNA) to MTC: the first one linked *MALAT1* with sMTC and, in the other, our group determined some new lncRNAs in a small group of sMTC cases in fresh tissue (*RMST, FTX*, *IPW*, *PRNCR1*, *ADAMTS9-AS2* and *RMRP*). The aim of the current study is to validate such novel lncRNAs previously described by our group by using a larger cohort of patients, in order to discern their potential role in the disease. Here we have tested three up-regulated (*RMST, FTX*, *IPW*) and one down-regulated (*RMRP*) lncRNAs in our samples (formalin fixed paraffin embedded tissues from twenty-one MEN2 and ten sMTC patients) by RT-qPCR analysis. The preliminary results reinforce the potential role of *RMST, FTX*, *IPW* and *RMRP* in the pathogenesis of MTC.

## Introduction

Thyroid cancer is the most common endocrine cancer, with an increasing overall incidence in recent decades, although it only accounts for 1% of all malignant tumors. It is divided into several types and histological subtypes according to the cells from which the tumor derives, with different characteristics and prognoses. Only 2–5% of cases of thyroid cancer are derived from parafollicular cells and this type is called medullary thyroid cancer (MTC) [1, 2] and occurs in both familial (ORPHA:99,361) and sporadic forms (ORPHA:1332). Approximately 20% of patients develop distant metastases, which makes MTC an aggressive and rare cancer incurable nowadays as this cancer does not respond to radiotherapy or chemotherapy (13.4% all thyroid cancer mortality) [3]. About 75% of all MTCs are believed to be sporadic (sMTC), whereas the remaining 25% correspond to inherited cancer syndromes known as Multiple Endocrine Neoplasia type 2 (MEN2). MEN2 includes 3 clinically differentiable types: MEN2A (ORPHA:247,698), MEN2B (ORPHA:247,709) and familial thyroid cancer (FMTC, ORPHA:99,361) [4, 5], which present MTC as a common feature and have been defined based on presence or absence of hyperparathyroidism, pheocromocytoma and other additional characteristic clinical features. *RET* proto-oncogene alterations play a crucial role for thyroid cancer development [3]. Different mechanisms of *RET* activation, gene rearrangements and point mutations characterize MTC, representing a very strong factor for its poor prognosis [6, 7]. More than 100 gain-of-function *RET* mutations have been reported in patients with MTC, including germline mutations (in patients MEN2 with hereditary disease) and somatic mutations (in patients with sporadic disease) [8]. More than 95% of MEN2 cases have germline mutations in exons 5, 7, 8, 10, 11, 13, 14, 15 and 16 of the *RET* proto-oncogene, which lead to a gain of function of the receptor. In the case of MEN2A, 98% of patients have mutations grouped within a hot-spot that corresponds to five cysteine codons present in the extracellular domain of the protein (codons 609, 611, 618, 620 and 634) [9]. Approximately 87% of MEN2A mutations affect to codon 634 of *RET*, and the p.Cys634Arg mutation has been found in more than 50% of cases [9]. In Spanish MEN2A patients, the p.Cys634Tyr mutation is more prevalent, which suggests a founder effect [10–12]. Biochemical studies on mutated proteins in cysteine codons indicate that these mutations lead to a constitutive activation of the metabolic pathways of RET signaling [13]. Regarding MEN2B, 95% of patients have a single mutation in exon 16 (p.Met918Thyr) that causes a conformational change in the intracellular tyrosine-kinase 2 binding pocket and allows for constitutive kinase activation in the absence of dimerization, as well as altered substrate binding. However, in about 5% of familial cases with “MEN2-like” presentation patients, the genetic cause of the disease is unknown.

Nowadays, MTC patients have limited treatment options for metastatic disease. Therefore, the exploration of new mechanisms implicated in the onset of thyroid cancer as well as new therapeutic targets is crucial to improve treatment. This perspective makes epigenetics an interesting area of research [14]. In this sense, many epigenetic processes have been implicated in thyroid tumorigenesis of MTC, such as ncRNA deregulation, especially microRNAs [15, 16], although it is difficult to identify a good biomarker of the disease [16]. Regarding long non coding RNA (lncRNA), there are two studies describing them in association with sMTC [17, 18]. However, no studies have been performed on MEN2 syndrome patients and lncRNAs. The broad term lncRNA indicates a class of non-coding RNA transcript of minimum 200 nucleotides in length, bigger than miRNAs with about 21–25 nucleotides in length. They have gained broad attention in recent years as new players in transcriptional, epigenetic, or post-transcriptional regulation of gene expression [19].

The aim of the present study is based on previous results from our group [18]. In that study, performed in tumoral and non-tumoral paired fresh frozen tissues from sMTC patients, and we have detected some lncRNAs already associated with thyroid cancer (GAS5, MALAT1, MEG3, PTCSC1, PTCSC3, H19), while some new were described (ZFAS1, RMST, SNHG16, FTX, IPW, ADAMTS9-AS2, PRNCR1 and RMRP). Thus, we aimed to amplify the cohort of patients, to validate such results. It is worthy to mention that ZFAS1 and SNHG16 were linked to papillary thyroid cancer after our publication [20–22] and that is the reason why we did not continue performing any additional study with them.

Therefore, unlike other previous assays, we have analyzed different lncRNA on both sMTC and MEN2 patients to find the link among their altered expression and their role on such manifestations, for the first time.

## Materials and methods

### Patients and tissue samples

From thirty-one MTC patients undergoing surgical resection, including both twenty-one MEN2 (twenty MEN2A and one MEN2B) and ten sMTC patients, medullary thyroid tumor tissues (FFPE, formalin fixed paraffin embedded tissues) and their corresponding adjacent non-tumor thyroid tissues were obtained. All the clinical data are compiled in Additional file 1: Supplementary Table 1.

**Table 1:** Aberrant lncRNAs in human sMTC and MEN2 FFPE tissues: data obtained by qRT-PCR (7500HT Taqman system) with RT^2^Sybr®Green Rox^TM^qPCR Mastermix using the cDNA obtained from the RNA isolated from the FFPE tissues of our cohort of patients

lncRNA	Genetic background	Fold change	Standard Error of the Mean (SEM)	Expression	T student test
*RMRP*	MEN2	0.3655	0.0749	Down-regulated	4.8358E−05
	sMTC	0.3025	0.1262		0.002
*FTX*	MEN2	6.2810	1.1032	Up-regulated	0.0007
	sMTC	6.8007	0.5953		0.0017
*IPW*	MEN2	13.5066	4.5254	Up-regulated	0.0139
	sMTC	18.4424	3.2108		0.0091
*RMST*	MEN2	3.7659	0.9464	Up-regulated	0.0177
	sMTC	2.1200	0.1227		0.0489

### RNA extraction from FFPE tissues and cDNA synthesis

RNA was extracted from all FFPE tissue specimens from MTC and MEN2 patients. Ten slides (ten µm each) were manipulated with the MasterPure™ Complete DNA and RNA Purification Kit (Lucigen) following manufacturer’s instructions. Briefly, we placed 30 mg of 10 µm thick paraffin sections in a tube with Proteinase K and Tissue and Cell Lysis Solution to mix. After 30 min at 65 °C (vortex and ice), we added MPC Protein Precipitation Reagent to pellet the debris by centrifugation (4 °C, 10 min, 10,000×*g*). The supernatant was mixed with isopropanol and rinsed twice with 70% ethanol to finally resuspend the total nucleic acids in TE Buffer. The contaminating DNA was removal using the DNAse I solution, MPC Protein Precipitation Reagent and different centrifugations. The resultant pellet with the purified RNA was resuspended with TE Buffer.

The RNA was quantified by Nanodrop (Invitrogen) and 1 μg of total RNA was reverse transcribed into cDNA using PrimeScript RT Reagent Kit (TaKaRa). Finally, RT^2^Sybr®Green Rox^TM^qPCR Mastermix (Qiagen) was used to determine lncRNA expression levels, using *Beta Actin* as reference gene.

### qRT-PCR

All the reactions were carried out in triplicate. All the data were analyzed by Applied Biosystems software and the relative expression levels of lncRNAs were determined by the Equation  2^−ΔΔCt^. The qRT-PCR was performed at 7500 Fast Real Time PCR System (Applied Biosystems). Primers used for *FTX*, *IPW* and *RMST* were the same used into SYBR®Green qPCR assays (RT^2^ lncRNA PCR Array, Qiagen) in the previous study. For *RMRP* and *Beta-Actin,* the primers used were KiCqStart® SYBR® Green Primers. All primer sequences are available under request.

Annotation analysis was performed using DAVID Bioinformatics Resources v6.8 online tools (http://david.abcc.ncifcrf.gov/).

### Statistics

Student’s *t* test was used to analyze lncRNA expression data collected from qRT-PCR.

Two-tailed t-test was used to analyze differences between tumor tissues and their corresponding adjacent non-tumor thyroid tissues. A P < 0.05 was considered as a statistically significant difference. Data are expressed as means ± standard error of the mean (SEM).

## Results

### Expression of lncRNAs analyzed

A total of seven lncRNAs were selected to be further studied in the current study: four of them were up-regulated (*RMST* or rhabdomyosarcoma 2 associated transcript), *FTX* or FTX transcript and XIST regulator), *IPW* or imprinted in Prader-Willi syndrome) while other three lncRNAs were down-regulated (*PRNCR1* or Prostate Cancer Associated Non-Coding RNA 1, *ADAMTS9-AS2* or Antisense RNA 2 and *RMRP* or RNA component of mitochondrial RNA processing endoribonuclease). Unfortunately, neither from *PRNCR1* nor *ADAMTS9-AS2* expression was detected in FFPE samples, and thus they were eliminated from the study. Then, the lncRNAs finally analyzed were *RMST*, *FTX*, *IPW* and *RMRP*.

**Figure 1:**
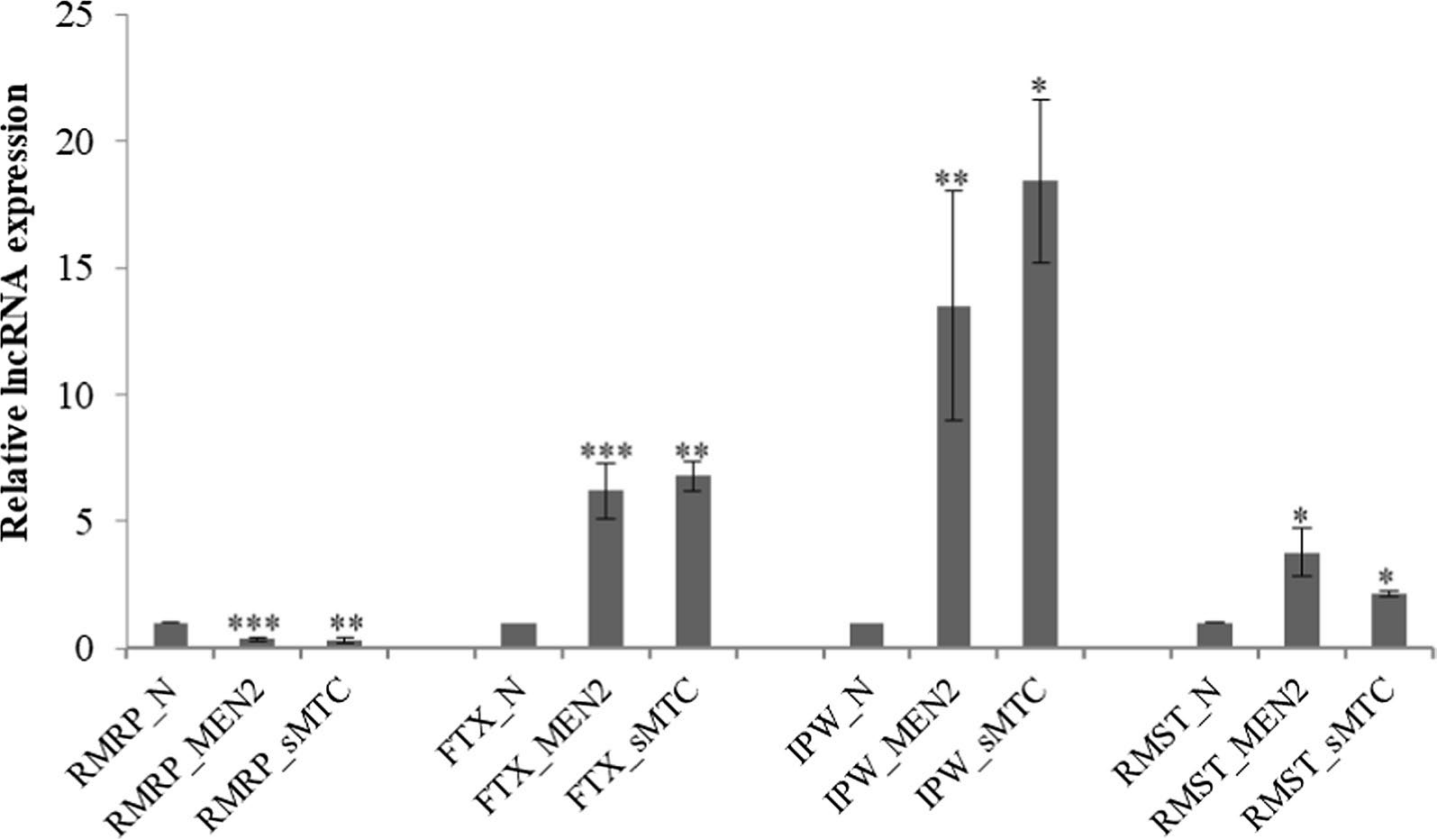
The expression levels of *IPW*, *RMRP*, *RMST* and *FTX *both in MEN2 and sMTC: This assay was performed by qRT-PCR in medullary thyroid tumor tissues (formalin-fixed paraffin embedded, FFPE) and their corresponding adjacent non-tumor thyroid tissues. Data represent the mean ± SEM from three independent experiments. *P < 0.05; **P < 0.01; ***P < 0.001. N= normal; sMTC=sporadic medullary thyroid cancer; MEN2= Multiple Endocrine Neoplasia type 2.

We used thirty-one formalin-fixed paraffin embedded (FFPE) tissues due to the difficulty to obtain fresh frozen tissue from both sMTC and MEN2 patients. The specimens included twenty-one patients with develop MTC due to a MEN2 syndrome and ten with sMTC (Additional file1: Supplementary Table 1). After analyzing the selected lncRNAs by qRT-PCR, we obtained a significant fold change (or log2 ratio) for the up-regulated lncRNAs: *RMST* (log2 = 11,359), *FTX* (log2 = 6,281), *IPW* (log2 = 14,616) and for the down-regulated *RMRP* (log2 = 0,338) (Table 1 and Fig. 1), which fits with the results obtained in our previous study performed on fresh sMTC tissue.

The functional annotation analysis of these lncRNA was performed based on their gene ontology categories (GOs) (Table 2). In either case, there is still little information about these lncRNAs to date [20–25].

**Table 2:** Gene ontology (GO) functional annotation analysis: annotation analysis was performed using DAVID Bioinformatics Resources v6.8 online tools (http://david.abcc.ncifcrf.gov/)

lncRNA	Species	GO Term	ORPHA Number
*RMST*	–	–	–
*FTX*	Rattus norvegicus	GO:0,006,325 ~ chromatin organization GO:0,010,468 ~ regulation of gene expression GO:0,044,030 ~ regulation of DNA methylation GO:1,900,095 ~ regulation of dosage compensation by inactivation of X chromosome	
	Mus musculus	GO:0,006,325 ~ chromatin organization GO:0,010,468 ~ regulation of gene expression GO:0,044,030 ~ regulation of DNA methylation GO:1,900,095 ~ regulation of dosage compensation by inactivation of X chromosome	
*IPW*	–	–	–
*RMRP*	Homo sapiens		ORPHA:175 ~ Cartilage-hair hypoplasia ORPHA:1838 ~ Metaphyseal dysplasia without hypotrichosis ORPHA:93,347 ~ Anauxetic dysplasia
	Mus musculus	GO:0,001,701 ~ in utero embryonic development	

(–) nothing described yet

## Discussion

Upregulated and downregulated lncRNAs, as post-transcriptional regulators of genetic expression, in different histological types of thyroid cancer, have been described [3, 6, 26]. Particularly, in MTC very few studies have been performed in this emerging field, despite the relevance of using lncRNAs as biomarkers in thyroid cancer diagnosis and prognosis, as well as their potential association with common genetic changes associated with thyroid cancer [17, 18]. As we previously mentioned, one study associates *MALAT1* as pro-oncogenic lncRNA in sMTC and the other one, published last year by our group, detected some dysregulated lncRNAs in sMTC patients (*RMST, FTX, IPW, PRNCR1, ADAMTS9-AS2* and *RMRP*). In addition, any study has linked lncRNAs with MEN2 syndrome to date. Thus, to our knowledge, this is the first study analyzing different lncRNAs (*RMST*, *FTX*, *IPW* and *RMRP*) and its role in MTC development, either in sMTC and MEN2 syndrome patients.

Briefly, *RMST* has been recently linked to cancer by its modulation of DNA methylation [27]. In addition, it has been described in hepatocellular [28] and endometrial cancers [29]. It functions in neurogenesis by helping in the association of Sox2 transcription factor to its target promoters (provided by RefSeq, Dec 2017). Some members of the SOX family are overexpressed in thyroid cancer [30]. In addition, SOX2 has been linked to Central Nervous System tumours as glioblastoma [31]. Such kind of tumours have been detected in MEN2A patients [32].

In this study, we have detected a significant upregulation of 3.7 fold (MEN2) and 2.1 fold (sMTC) in comparison with the normal tissue of such patients. These outcomes would indicate that maybe *RMST* is more implicated in familial cases than in sporadic ones from MTC. Thus, based on the literature and in our own study, a potential role for *RMST* in MTC pathogenesis, especially in familial cases, should be further analyzed.

*RMRP* encodes the RNA component of mitochondrial RNA processing endoribonuclease, which cleaves mitochondrial RNA at a priming site of mitochondrial DNA replication (provided by RefSeq, Mar 2010). It has been described that miR-675 directly targets MAPK1, and inhibits the oncogenicity of thyroid cancer while it is sponged by *RMRP* [33]. Moreover, mutations in *RMRP* have been associated with a spectrum of autosomal recessive skeletal dysplasias classified as cartilage-hair hypoplasia (CHH, ORPHA:175), whose patients may develop adult-onset immunodeficiency or malignancy such as thyroid cancer [34]. In the current work, we have found a significant downregulation of *RMRP* of a 63.49% (MEN2) and 69.75% (sMTC), which give us an indication that this molecule could be implicated in MTC with a little more incidence in sporadic cases. Then, together with those previously mentioned studies, our results would complement its potential association into medullary thyroid cancer development.

LncRNA *FTX* was firstly identified in *XIST* gene locus and was dysregulated in many human cancers [15, 35, 36]. *FTX* is located upstream of *XIST*, within the X-inactivation center, and produces a spliced lncRNA which positively regulate the expression of *XIST*, which is essential for initiation and spread of X-inactivation (provided by RefSeq, May 2015). It has been described that *XIST* was significantly upregulated in thyroid cancer [37], through promotion of cell proliferation and invasion [23]. We found here that *FTX* is significantly upregulated (6.2 fold in MEN2 and 6.8 fold in sMTC), although there are little differences in the expression pattern of this lncRNA in both type of patients. Then, if FTX is upregulated, then *XIST* will be also upregulated, potentially promoting the onset or develop of this pathology.

*IPW* is a non-protein coding gene exclusively expressed from the paternal allele, maybe playing a role in the imprinting process. Mutations in this gene are associated with Prader-Willi syndrome (PWS; ORPHA:739) [provided by RefSeq, May 2010]. Its overexpression in the critical region of the PWS locus, which regulates the DLK1-DIO3 region, resulted in chromatin modifications, leading to a subset of PWS phenotypes [21]. Subjects with PWS need to regularly control their thyroid function, because they present metabolic and endocrine complications, such as hypothyroidism, which would reinforce the implication of *IPW* into thyroid cancer [38, 39]. It is important to highlight that in this study we have detected a significant upregulation of IPW either in MEN2 (13.5 fold) and sMTC (18.4 fold). Then, IPW seems to have a role into MTC and especially in sporadic cases.

One of the major limitations of our study is the use of FFPE tissues instead of fresh tissue, because rare tumours are rarely available. That was the reason why in our previous study we only can account with four fresh tissue of sMTC patients. Another limitation is that, at this point, we have only confirmed the group of lncRNAs through performing a qRT-PCR. Thus, different further studies should be made to validate the obtained results, such as in situ hybridization to detect differences of lncRNA expression among normal and tumoral adjacent part of the tumour, studies in cell lines of MTC, such as TT cells (invasion, proliferation, silencing/overexpression of lncRNA target, apoptosis, cell cycle assays …) [8, 40].

In summary and being conscious that further functional analyses are needed, we propose *RMRP* and *RMST* as potential candidates for a deeper knowledge in MEN2 patients and *FTX* and *IPW* in sMTC.

Considering our results and all the information previously published, this preliminary study has allowed us to identify four potential lncRNAs as suitable novel biomarkers for these rare diseases.

## Conclusions

Very few studies have been performed in the rising field of lncRNAs and medullary thyroid cancer. Based on previous results, we have validated four lncRNAs as potential diagnostic biomarkers in medullary thyroid carcinoma. Further future molecular analyses will be required to deep into their exact role in the pathology, focusing on their clinical validation and viability for thyroid cancer therapy in those patients who fail conventional therapy.

## Supplementary Information

**Additional file 1. Supplementary Table 1** Clinicopathological features of the enrolled patients in this study: TNM: tumor-node-metastasis; T: size or direct extent of the primary tumor; Tx: tumor cannot be assessed; T0= no evidence of tumor; T1, T2, T3, T4: size and/or extension of the primary tumor; N: degree of spread to regional lymph nodes; Nx: lymph nodes cannot be assessed; N0: no regional lymph nodes metastasis; N1: regional lymph node; N2: tumor spread to an extent between N1 and N3; metastasis present; at some sites, tumor spread to closest or small number of regional lymph nodes; N3: tumor spread to more distant or numerous regional lymph nodes; M: presence of distant metastasis; Mx: metastasis cannot be assessed; M1: metastasis to distant organs (beyond regional lymph nodes); p: stage given by histopathologic examination of a surgical specimen; CEA: carcinoembyronic antigen; PET/CT scan: computed tomography scan; MIBG: scan is a nuclear medicine scan that involves an injection of a radioactive medication (radiopharmaceutical) called iodine-123 meta-iodobenzylguanidine.

## Data Availability

Data sharing is not applicable to this article as no datasets were generated or analyzed during the current study.

## Abbreviations

lncRNAs: Long non-coding RNAs
MTCs: Medullary thyroid cancers
sMTC: Sporadic medullary thyroid cancer
MEN2: Multiple Endocrine Neoplasia type 2
FMTC: Familial thyroid cancer
RET: Rearranged during transfection
RMST: Rhabdomyosarcoma 2 associated transcript
FTX: FTX transcript and XIST regulator
IPW: Imprinted in Prader-Willi syndrome
PRNCR1: Prostate Cancer Associated Non-Coding RNA 1
ADAMTS9-AS2: Antisense RNA 2
RMRP: RNA component of mitochondrial RNA processing endoribonuclease
GOs: Gene ontology categories
SOX family: SRY-related HMG-box genes
FFPE: Formalin fixed paraffin embedded tissues
qRT-PCR: Quantitative reverse transcription polymerase chain reaction
