# Supplementary material for: RMRP, RMST, FTX and IPW: novel potential long non-coding RNAs in medullary thyroid cancer
Source: Orphanet J Rare Dis. 2021 Jan 6;16:4. doi: 10.1186/s13023-020-01665-5 (PMC7789680; doi:10.1186/s13023-020-01665-5)
Supplement: Supplementary file 1 — Additional file 1. Supplementary Table 1 Clinicopathological features of the enrolled patients in this study: TNM: tumor-node-metastasis; T: size or direct extent of the primary tumor; Tx: tumor cannot be assessed; T0= no evidence of tumor; T1, T2, T3, T4: size and/or extension of the primary tumor; N: degree of spread to regional lymph nodes; Nx: lymph nodes cannot be assessed; N0: no regional lymph nodes metastasis; N1: regional lymph node; N2: tumor spread to an extent between N1 and N3; metastasis present; at some sites, tumor spread to closest or small number of regional lymph nodes; N3: tumor spread to more distant or numerous regional lymph nodes; M: presence of distant metastasis; Mx: metastasis cannot be assessed; M1: metastasis to distant organs (beyond regional lymph nodes); p: stage given by histopathologic examination of a surgical specimen; CEA: carcinoembyronic antigen; PET/CT scan: computed tomography scan; MIBG: scan is a nuclear medicine scan that involves an injection of a radioactive medication (radiopharmaceutical) called iodine-123 meta-iodobenzylguanidine. [file 13023_2020_1665_MOESM1_ESM.docx]

**Supplementary table 1**

| **Nº patient** | **Gender** | **Age at diagnosis (years)** | **Diagnosis** | **Type of inheritance** | ***RET* mutation** | **Histological valoration of samples** | **Medical history** | **Tumor/metastasis description** | **Hormones** | **Radiological studies** |
| --- | --- | --- | --- | --- | --- | --- | --- | --- | --- | --- |
| 1 | Female | 24 | Multifocal medullary thyroid carcinoma. TNM: Ca medular de tiroides T1N0M0 | MEN2A | C634Y | Normal: Non neoplastic thyroid ganglia + less than 5% of tumor; Tumoral: 70-80% tumor + stroma + non neoplastic thyroid | 1999: Bilateral pheochromocytoma. 2000: Total thyroidectomy. Post-surgical hypothyroidism and adrenal insufficiency | Multifocal medullary carcinoma with a focus greater than 0,8 cm with partial expression of thyroglobulin + 3mm papillary thyroid carcinoma that contacts the resection border + diffuse lymphoid thyroiditis. | Thyroglobulin: positive. Calcitonin: positive and increasing since 2012. | No data available |
| 2 | Male | 41 (dead 7 years after diagnosis) | Medullary thyroid carcinoma. TNM: T4N1M1 | Sporadic | No | Normal: Non neoplastic thyroid; Tumoral: 50% tumor + stroma + less than 10% non neoplastic thyroid | 2002: Total thyroidectomy with persistent high calcitonin. 2004: Right cervical jugular-carotid and supraclavicular lymph node emptying. 2005: New right lymph node emptying with pathological anatomy of medullary thyroid carcinoma and skeletal muscle metastasis. 2006: Neoplastic progression at the cervical, paratracheal, bone and pulmonary levels.  2007: Cervical radiotherapy and neoplastic progression at cervical and pulmonary level.  2008: Neoplastic progression at liver level.  2009: Radiotherapy for left sacroiliac bone metastases and L4-L5. | Medullar thyroid carcinoma classic form in right thyroid lobe (1,2 cm of max diameter). | 2004 and 2005: Persistence of high calcitonin and diarrheal syndrome. Thyroglobulin: negative. Calcitonin: positive | CT scan with contrast (abdomen, pelvis, neck and chest)  2006: Right hypervascular laterocervical lymphadenopathy, with a polylobulated image of possible recurrence. Bilateral, millimeter lung metastases. Skeletal metastases. 2009 (year of death): Extensive neoplasm in thyroid, metastases in neck, mediastinum, hilar, lung, liver and perihepatic, renal and retroperitoneal lymph and bone. |
| 3 | Male | 50 | Medullary thyroid carcinoma with melanin pigment. TNM: T1N0M0 | MEN2A | C634Y | Normal: Non neoplastic thyroid + ganglia Tumoral: 70% tumor + stroma + less than 10% non neoplastic thyroid | 2002: Total thyroidectomy. No revision since 2006. | Total thyroidectomy piece of 14 gr and 5,5x4 cm, with the right lobe being the greater lobe. Nodules do not exceed the capsule that surrounds them. Rest of the parenchyma without relevant alterations. | Calcitonin: high levels Maleteined. Intensive positivity in tumor areas. Thyroglobulin: Positivity in extra-follicular follicular areas. | 2005: PET/CT scan and neck ultrasound:negative. Few laterocervical ganglia with a reactive-inflammatory appearance. |
| 4 | Female | 18 | Medullary thyroid carcinoma. TNM: T2N0M0 | MEN2A | C634R | Normal: Non neoplastic thyroid; Tumoral: 70% tumor + stroma + less than 10-15% non neoplastic thyroid | Family history: Father and one sister with MEN 2A syndrome. 2003: Total thyroidectomy: Medullary thyroid carcinoma operated twice with emptying of levels II, III, IV and V left. Left recurret lymphadenectomy). Metastasis of medullar carcinoma in nine of fourteen ganglios, with extraganglionar extension (largest metastasis of 0,5cm). A parathyroid gland with adenoma of 0,7cm. 2009: Bilateral pheochromocytoma operated on by laparoscopic bilateral adrenalectomy. | Total thyroidectomy piece (25 gr), the external surface is regular with a well capsule preserved. The greater lobe is the right lobe in which there is a limited tumor when serially cut. A serial cut of the left lobe reveals a multinodular tumor of the same features. The largest node is 1 cm. The right adrenal gland: (5,3x3 cm) shows a serial nodule, well delimited that measures 1,7 cm of expansive edges, and that does not exceed the capsule or periglandular fat. The left adrenal gland (6,5x3 cm) with two independent nodules teeth: the 1st of them that does not seem to exceed the supra capsule renal. | Persistance of elevated calcitonin. | 2012 Neck ultrasound: A left paratracheal nodule suspected of tumor recurrence. 2016: This left paratracheal nodule persists, remaining stable in size (max diameter of 5,8 mm). Other nodules of left paratracheal location have appeared. With well-defined margins, hypoechogenic and highly vascularized. A new, small (2 mm) nodule is also visualized at the right paratracheal level that was not visible in the previous study. |
| 5 | Male | 35 | Medullary thyroid carcinoma. TNM: T2N0M0 | MEN2A | C634R | Normal: Non neoplastic thyroid; Tumoral: 80% tumor + stroma + 10% non neoplastic thyroid | Family history: Mother, uncles and several brothers with MEN 2A syndrome. 2004: Total thyroidectomy: Medullar thyroid carcinoma, conventional form, with multi-central and bilateral affection. | Total thyroidectomy piece (7x6x3 cm; 50 gr). The external surface is violet and irregular. The lobe or right: there is a 1,3 cm, whitish neoformation, with irregular and well-defined edges of the thyroid parenchyma. Apparently, the lesion does not contact the thyroid capsule. In left lobe there is a new formation, 0.4 cm which does not contact with thyroid capsule. | 2004: Calcitonin: Positive in adjacent neoplastic and parafollicular cells. Thyroglobulin: Positive in follicular structures. 2009 (last analytical control): normal catecholamines and negative calcitonin. | No data available. |
| 6 | Female | 57 | Medullary thyroid carcinoma (non-encapsulated variant). TNM: T1N0M0 | MEN2A | C634R | Normal: Non neoplastic thyroid; Thyroid: 70% tumor + stroma + 10-15% non neoplastic thyroid | 2004: Medullary thyroid carcinoma, without disease data. 2010: Bilateral adrenal hyperplasia and right adrenal pheochromocytoma (right suprarenalectomy). | Total thyroidectomy piece (20 gr; 7 x 2,5 cm). Externally no macroscopic features. When cut, an apparent nodular tumor is distinguished in the left lobe, not encapsulated. On the right lobe, it was identified a tumor of 0,3 cm of similar characteristics. Hyperplasia of parafollicular cells. Right jugular chain contains irregular fragments (volume< 0.1 cc). Left jugular chain contains irregular fragments (volume<0.05 cc).  The neoplasm presents: - Less than 20 mitosis/absence of necrosis/amyloid deposits. | Calcitonin: Positivity in adjacent neoplastic and parafollicular cells. Chromogranin: Positivity in adjacent neoplastic and parafollicular cells. Bcl 2: Positivity in neoplastic cells. Thyroglobulin: Positive in follicular structures. TTF-1: Negative. | MIBG scintigraphy: uptake in both adrenals.  Magnetic Resonance Imaging: a 1 1 cm nodule in the right adrenal. Pheochromocytoma 0,5 cm in diameter. |
| 7 | Male | 37 | Medullary thyroid carcinoma | MEN2A | C634Y | Normal: Non neoplastic thyroid; T: 60% tumor + stroma + non neoplastic thyroid; | 2004: Total thyroidectomy: Medullary thyroid carcinoma with millimeter tumor focus. No post-surgical complications. | Thyroid gland (10hr; 5x3,5x1,5 cm). No macroscopic alterations. Two whitish nodules are identified, with scalloped edges and displacements (0,9 and 1,1 cm). The nodules do not appear to contact the thyroid surface. | 2018: Increased value of Calcitonin. | 2018/2019 Neck ultrasounds: no alterations in the theoretical location of parathyroid. No cervical lymphadenopathies of pathological appearance. SubMaledibular and parotid glands with normal characteristics. |
| 8 | Female | 40 | Medullary thyroid carcinoma. Metastasis in eleven lymphoid ganglia. TNM: T2N1M0 | MEN2A | C634Y | Normal: Non neoplastic thyroid + ganglia + less than 5% tumor; Tumoral: 60% tumor + stroma + non neoplastic thyroid | Family history: two maternal cousins with mama carcinoma. | Total thyroidectomy + central lymph node emptying: multicentre medullary carcinoma with focus greater than 1,2 cm that does not exceed capsule +metastasis in 11/17 lymph nodes with extracapsular extension in the right chain and 0/7 in the left chain. | 2005: Asymptomatic, normal examination. 2007: Calcitonin in progressive decline after thyroidectomy. 2008-2011: Calcitonin stable; CEA high and stable. 2020: Calcitonin with moderate increase. | Neck ultrasounds: 2009: Two nonspecific lymph nodes (5 and 6 mm) in the right chain.  2016: Solid hypoechoic nodule, oval (8,8 x 3,8 x 5,2 mm) in the right occipital chain without calcifications, cystic changes and normal. 2018: Total thyroidectomy, without evidence of abnormalities in the surgical bed. A small entity lymph node distributed homogeneously. Persistent non-localized disease.  2020: In the lower right jugulocarotid chain: a heterogeneous nodule (7 x 8 mm), suggestive of adenopathy. |
| 9 | Female | 43 | Medullary thyroid carcinoma. TNM: stage pT1, N0 | MEN2A | C634Y | Normal: Non neoplastic thyroid  Tumoral: 30-40% tumor + stroma + non neoplastic thyroid | 2005: Total thyroidectomy + central lymph node emptying. Patient with diffuse large B-cell lymphoma / follicular lymphoma grade IIIB (60% / 40%) with lymphadenopathy at the neck, supra and infraradiaphragmatic lymph nodes with lymphomatous infiltration of the spleen.  2017: Diffuse large cell lymphoma B / follicular lymphoma grade IIIB stage IV-B, IPI 3, AIPI 2, in remission. | 2014: Cervical polyadenopathy. Splenomegaly with multiple pathological focal lesions. All suggestive of malignancy Adrenalectomy:bilateral pheochromocytoma (2,2, cm), signs of hyperplasia medullary.  Total thyroidectomy piece (25 gr) and whose lobes are separated. The largest lobe (3,5 cm) and the serial cut shows multiple well-defined white nodules of different diameters. Scattered lesions over 50% of the cutting surface. The largest nodule is 4 mm. The smaller lobe shows the same pattern of multinodular tumor but standing out in the presence of a nodule (9 mm). The thyroid capsule is free of injury.  Absence of extra-thyroid extension. | 2005: Calcitonin and Chromogranin positive in neoplastic and hyperplastic C cells. | 2015: PET/CT scan with intravenous iodinated contrast: Neck: enlarged ganglion formations are seen affecting practically all laterocervical chains, highlighting the largest on the left side of 32 x 25 mm.  Thorax: enlarged ganglion formations at rib cage in axillary regions (2,5 cm, left side), at the mediastinal level.  Abdomen: Spleen enlarged with multiple focal lesions compatible with lymphomatous infiltration and liver a single focal lesion of undetermined low attenuation. In the pelvis, adenomegalies are also observed in both bilateral external iliac and inguinal chains. With a bone window, a moderate diffuse demineralization is observed. Vertebral crushing and altered attenuation at the L2 and L4 level. Slight degenerative vertebrodiscal disorders. |
| 10 | Male | 45 | Medullary thyroid carcinoma. TNM: pT1, pN1 | MEN2A | C634Y | Normal: Non neoplastic thyroid; Tumoral: 40% tumor + stroma + non neoplastic thyroid | 2007: Total thyroidectomy: multicenter spinal cord, focus greater than 1 cm in diameter. Transient post-surgical hypoparathyroidism. Left adrenal pheochromocytoma. Left adrenalectomy. Post-surgical hypoparathyroidism  2013: hyperplastic colon polyps.. | 2007: Total thyroidectomy piece (40 gr; 8x6x3 cm). Abundant brownish nodules up to 0,6 cm in diameter are isolated from a fibro-fatty tissue fragment. Two fragments of adenopathy on the left side.  Multicenter medullary carcinoma, located in both lobes, up to 1 cm in diameter, the largest. Without capsular invasion, extrathyroid extension or angioinvasion. Free resection margins. Twenty-nine lymph nodes, eight of them with metastasis from medullary carcinoma and absence of extranodal extension. Left adrenalectomy piece (25 gr; 7 x 3 cm) that at the serial cut shows at the medullary level a nodular formation (0,8 x 0,8 cm). Pheochromocytoma (1 cm). | Medullary thyroid carcinoma, persists with elevated calcitonin without localization. | 2007: PET/CT SCAN with contrast (neck): a left thyroid lobe that presents a hypodense nodular image in the lower pole and another larger one also in the left thyroid lobe. In laterocervical regions of the neck on the right side, a adenopathy of about 1 cm. There is another lymphadenopathy in the left jugular region. 2013 CT of the chest: Left adrenal lesion suggestive of pheochromocytoma in a patient with MEN 2A syndrome and elevated urine catecholamines.  2017: radiological stability compared to previous examination in 2013. Alterations compatible with partially calcified chronic hematoma lodged within the right sternocleidomastoid muscle at the supraclavicular region. Small right paratracheal nodular image in the thyroidectomy bed. 2020: Neck ultrasound with a high resolution multi-frequency linear probe (5-14 MHz). Right paratracheal ganglion (level VI) of 9 mm, with morphology within the limits of normality. Probable smaller bilateral paratracheal nodes. Partially calcified mass adjacent to the right mastoid sternocleid muscle that may correspond to organized hematoma. |
| 11 | Male | 62 | Medullary thyroid carcinoma. T2N0M0 | MEN2A | C630Y | Normal: Non neoplastic thyroid; Tumoral: 80-90% tumor + stroma | Family background: 1 daughter MEN 2A syndrome Personal history: diabetes mellitus type 2 and Medullary thyroid carcinoma without disease data. | 2014: Thyroidectomy piece (25 gr), partially torn at the level of the isthmus, showing a greater lobe of 4,5x3 cm and a smaller lobe of 4x2,5 cm. Thyroidectomy with right and left emptying): thyroid with bilateral and multifocal medullary carcinoma without extension to extrathyroid tissues, the largest node of which is 2 cm. | 2007: Calcitonin in progressive decline after thyroidectomy. | Thyroid ultrasound: 2.28x1,5x2,5 cm nodule located in the right lobe.  2014: Chest study within normal limits. |
| 12 | Female | 34 | Medullary thyroid carcinoma. T2N0M0 | MEN2A | C630Y | Normal: Non neoplastic thyroid; Tumoral: 80-90% tumor + stroma + less than 5% non neoplastic thyroid | Personal history:  2014: total thyroidectomy and central neck emptying thyroid with bilateral and multifocal medullary carcinoma, without extrathyroid extension.  Postsurgical hypoparathyroidism. Systemic lupus erythematosus with nephrotic syndrome possibly due to type 5 nephropsy. Leukolinfopenia. | Thyroid gland (20 g) with a 4x2 cm right thyroid lobe, an isthmus and a 3x1,7 cm left lobe. Smooth external surface, full capsule. When the right lobe is cut, a well-defined, whitish, multinodular lesion of 2,5 x 1,5 cm is identified that, macroscopically, is 0.1 cm from the thyroid capsule. When the left thyroid lobe was cut, a multinodular lesion measuring 0,7 x 0,5 cm. | 2008-2011: Calcitonin stable. CEA high and stable. | 2009: Neck ultrasound: two nonspecific lymph nodes of 5 and 6 mm.  2014: Chest X-ray: Cardiomediastinal silhouette, bronchovascular structures, lung parenchyma and rib cage structures without relevant alterations. |
| 13 | Male | 55 | Unifocal medullary thyroid carcinoma and papillary microcarcinoma (sporadic). TNM T2N1bM0 | Sporadic | No | Normal: Non neoplastic thyroid; Tumoral: 80% tumor + stroma + 10% non neoplastic thyroid | Personal history:  2014: Medullary sporadic thyroid carcinoma with 2 mm papillary microcarcinoma.  Neck: nodule of 1,5-2 cm of firm consistency. No palpable lymphadenopathy. Lung lesions compatible with stable granulomas in imaging test. | Total thyroidectomy piece (50 gr): thyroid with two malignancies: a unifocal and intrathyroid medullary carcinoma measuring 2,5 cm and a papillary microcarcinoma of 2 mm and a conventional histological type. Micrometastasis from medullary carcinoma in 1/10 lymph nodes isolated from left lymph node emptying; absence of tumor extension to extranodal tissues | Immunohistochemical study (thyroglobulin and calcitonin) confirms this diagnosis. hyperplasia of cells.  Uundetectable Calcitonin and CEA levels in blood. | 2014: PET/CT SCAN with contrast: Chest: Hypodense nodular lesions. No evidence of adenomegaly in axillary, mediastinal, or bronchopulmonary ganglion chains. Subcentimetric pulmonary nodules, the largest of 7 mm and others of very few millimeters. Calcified millimetric nodules in the right lung.  Abdomen: Liver of normal size, morphology and attenuation. Gallbladder and bile ducts without pathological findings. Colonic diverticulosis, with no signs of current complication.  2014: Thyroid ultrasound: Lower pole nodule of solid hypoechoic somewhat heterogeneous inside with well-defined hypoechoic halo with intranodular vascularization and measuring 2,12x1,90x2,30 cm. |
| 14 | Female | 38 | Medullary thyroid carcinoma and papillary thyroid carcinoma (folicular pattern, sporadic). T3N1bMx. | Sporadic | No | Normal: Non neoplastic ganglia; Tumoral: 80% tumor + stroma + lymphoid tissue | Family history: - Paternal grandmother with renal cell carcinoma (87 years). - No family history of Thyroid Cancer. - 5 healthy brothers. - Healthy 17-year-old daughter. Personal history: -Surgical interventions: myoma 3 years ago. -Sporadic mixed papillary and medullary thyroid carcinoma. Peritiroidal and periganglionic extension. Affected margin. Medullary lymphatic involvement. High risk. Persistent non-localized disease. Post-surgical hypoparathyroidism. Recurrent left vocal cord paralysis secondary to subtotal thyroidectomy.  2015: Total thyroidectomy and cervical lymph node emptying. | Total thyroidectomy piece (15 gr; 4.5x3.5 cm right lobe). Externally, surface without alterations. When cut: a homogeneous parenchyma with a 0.3 cm nodular formation and homogeneous translucent content, which appears to be colloid, is identified. Left lobe 4x2.5 cm. Externally, uneven surface. When cut, the entire parenchyma is identified, occupied by an irregular 2.5x1.7 cm mass with a yellowish-brown heterogeneous surface and covered with soft tissue consisting of muscle tissue. Left lateral emptying: Irregular fragment with several nodular formations, two of them stand out (3.5 and 3 cm), which when cut, in the largest one, identifies a heterogeneous parenchyma like that found in the left thyroid lobe. Right lateral adenopathy biopsy: 1.5 cm nodular fragment. Diagnosis: Thyroid with two malignancies: a medullary carcinoma and a conventional papillary carcinoma with a follicular pattern. Both neoplasms are unifocal and coincide in the 2.5 cm nodule in similar proportions, infiltrating extrathyroid soft tissues and reaching surgical resection margins. absence of prominent c-cell hyperplasia.  Metastasis of medullary carcinoma in five of twelve lymph nodes (0,5-2 cm), with extension to extranodal soft tissues).  Absence of lymph node metastases from papillary carcinoma.  Neoplasm-free lymph node. | Pre-surgery markers: CEA: 221 and Calcitonin: 1710 pg / ml.  2014-2017: Elevated calcitonin levels.  2018: decreased calcitonin levels. 2019: hormone levels stabilized. | 2014: CT with contrast IV of the abdomen: No pathological findings at the abdominal level. Liver with normal morphology and echogenicity.  CT chest and neck: Left thyroid nodule with foci of calcification and left laterocervical lymphadenopathy at lymph node levels III and IV: lymph node metastases.  A small nodule in right lobe (2,8 mm), undetermined. Two left-sided cervical suspicious lymphadenopathies.  2019: PET/CT SCAN with Contrast I.V. Face and neck: Radiological stability without changes at the level of the thyroidectomy bed or in the preexisting nodes. |
| 15 | Female | 54 | Multifocal medullary thyroid carcinoma. T1N0M0 | MEN2A | V804M | Normal: Non neoplastic thyroid; Tumoral: 60% tumor + stroma + non neoplastic thyroid | Personal history: Uterine myoma (myomectomy and partial removal of left ovary). No medication. Fully asymptomatic. 2015: Total thyroidectomy  Complications: Post-surgical hypocalcemia, some post-surgical hoarseness. | Thyroid gland: - multifocal and intrathyroid medullar carcinoma, with focuses between 0,1 and 0,6 cm, respecting the margins surgical surgery. presence of C cell hyperplasia. - conventional, unifocal and intrathyroid papillary microcarcinoma, measuring 0,1 cm and respecting surgical resection margins. - lymphocytic thyroiditis and hyperplastic node with oncocytic change (1cm). Total thyroidectomy piece (10 gr; right lobe 3x1,7 cm, left lobe 3x1,7 cm). The surface is smooth, with a violet-brown coloration and a soft consistency, without identifying nodular formations of interest. After the cuts, several well-defined nodular lesions (0,2-0,4 cm) in the right lobe, none of them in contact with the thyroid capsular surface. The left lobe also presents multiple, well-defined nodular lesions with a whitish-brown coloration (0,1- 0,3 cm), none of them in contact with the capsular surface. Three lymph nodes of 0,2 cm (largest). | Calcitonin and chromogranin positive in medullary carcinoma cells (negative in papillary microcarcinoma). | Neck ultrasound: Thyroid of normal size. A well-defined hypoechoic nodule, with a central cystic zone, with peripheral vascularization (1x0.5x1.35 cm). In lower pole hypoechoic nodule less than 1 cm in diameter and in the posterior middle third another small nodule of approximately 0,5 cm.  2016: no ultrasound findings that suggest the existence of tumor remains / recurrence in the thyroid bed. |
| 16 | Female | 68 | Multifocal medullary thyroid carcinoma (sporadic). Periganglionar metastasis. TNM: Not available | Sporadic | No | Normal: Non neoplastic thyroid; Turmoral: 90% tumor + stroma | Family history: Father lung carcinoma. Personal history: Irritable colon. Total thyroidectomy and bilateral central lymph node emptying and left modified radical cervical emptying Thyroid cancer with left lateral lymph node metastasis. | Thyroidectomy and central emptying: piece made up of a thyroid, 4x2 cm of major axis, which, when cut, shows a cystic lesion of 0,5 cm in the lower hemoglobe and the rest consisting of homogeneous violet tissue without alterations. In the left lobe we observed a tumor mass (3 cm), and an irregular fragment of 2 cm of tissue with a musculoskeletal appearance. Diagnosis: thyroid with 3 cm left lobe medullar carcinoma with marked extension to perithyroid tissues. Metastasis of the medullar carcinoma in 7/13 ganglios. Lateral emptying: 6 cm medullar carcinoma metastasis, with marked extension to periganglionar tissues. | Imunohistochemical study: the carcinoma is positive with calcitonin and negative with thyroglobulin. Calcitonin has risen but very little for the growth experienced by the bone lesion. | Neck Ultrasound: - Three nodules, two of them cystic and one solid, measuring 14,4x6,9 mm. - A hypoechogenic nodule, 20x15,1x33mm and with spiculated and irregular edges and with calcifications, central vascularization. - Left III lymph node chain: an adenopathic bundle (2 cm), irregular and with apparent calcifications.  Cytological findings of carcinoma with marked signs of cellular pleomorphism, probably undifferentiated papillary carcinoma, without being able to rule out medullary carcinoma. |
| 17 | Male | 34 | Medullary thyroid carcinoma. T1N0M1 | MEN2A | C634Y | Normal: Non neoplastic thyroid; Tumoral: 40-50% tumor + stroma + non neoplastic thyroid | Personal history: 1995: Medullary thyroid carcinoma. Persistence of disease Right adrenal pheochromocytoma. Liver metastases from medullary thyroid carcinoma (2015). Transrectal prostate biopsy with the pathological diagnosis of Prostate Adenocarcinoma with Gleason 6 (3 + 3) in a cylinder. | Suspected pheochromocytoma and liver metastases from medullary thyroid carcinoma in segments VI-VIII. Diagnosis: Right adrenal gland and liver (excision): -Right adrenal gland: - Medullary hyperplasia. -hepatic metastases segments VI-VII-VIII  - gallbladder without relevant alterations. | Calcitonin levels in the last year of 144-130 and 127 pg / ml with negative imaging tests. Normal urinary catecholamine levels until 2004, at which time an elevation of urinary metanephrines was observed. Discrete elevation of calcitonin but with values like those of 2010.  2015: Calcitonin stable, but CEA on the rise. | 2011: PET / CT scan: Thoracoabdominal with Fludeoxyglucose-F18 Medullary thyroid. No notable findings on structural images.  2014: Neck Ultrasound Changes after cervical thyroidectomy without evidence of glandular remains in the surgical bed. No signs of metastatic disease in the different cervical ganglion chains. Calcified atheroma plaques in carotids without evidence of lumen stenosis.  2016: Chest and Abdomen CT: No signs of tumor extension of the treated neoplasms. Stable liver metastasectomies. Right adrenalectomy. Atrophic right kidney. Left renal cortical cyst. Decreased intervertebral space L5-S1 with sign of empty disc. 2016: Ultrasound of the Abdomen: A small cyst in segment VII. Prostate of normal size, with echogenic foci inside. Right kidney decreased in size. The avascular cyst in segment VII is identified. A pathological tracer deposit located in segment VIII of the liver was observed, which was 13 mm in size and had a maximum SUV of 4.14 (normal liver SUV: 2.2) and which was suggestive of malignancy. There is another small tracer accumulation in the left cervical region, which seems to correspond to a lymph node. This finding is suggestive of malignancy. 2016: Neck ultrasound: Solid thyroid nodule with irregular contours and with prominent internal vascularization, suspected of malignancy. Adenopathy at right level IV, to assess metastatic involvement of the thyroid injury. |
| 18 | Male | 36 (previously included as fresh tissue in our Maleuscript 2019) | Medullary thyroid carcinoma (sporadic). T3N1M0 | Sporadic | No | Normal: Non neoplastic thyroid; Tumoral: 70-80% tumor + stroma + less than 10% non neoplastic thyroid | Family history: Father: Leukemia Mother: Rheumatoid arthritis. 3 Brothers. 1 brother with heart surgery. | 2016: Total thyroidectomy with central right cervical emptying with large supraclavicular adenopathy (2,5 cm medullary cancer, unifocal and with extraglandular extension. Right central emptying: Irregular fragment of fibroadipose tissue (3x2 cm), in which three lymph nodes (0,5-1,5 cm). Several irregular fragments of fibro-fatty tissue (4x3cm, the largest), isolating multiple lymphoid nodules clearly metastatic. Two lymph nodes (1,7 cm, the largest), of increased consistency. | Initially with normal Calcitonin and currently stable since August / 2019. | 2015 Neck Ultrasound: Solid thyroid nodule with irregular contours and with prominent internal vascularization, suspected of malignancy. denopathy at right level IV. Lymph nodes with morphological characteristics and normal size in the rest of the laterocervical ganglion chains. Supraclavicular region: infiltration by papillary carcinoma. 2020 Neck Ultrasound: No images that suggest remains or tumor recurrence in the surgical bed. |
| 19 | Male | 11 | Multifocal medullary thyroid carcinoma. T1N0M0 | MEN2B | M918T | Normal: Non neoplastic thyroid; Tumoral: 70-80% tumor + stroma +non neoplastic thyroid | Family history: Maternal aunt with goiter. Personal history: Genetically confirmed MEN 2B, predisposing to medullary thyroid carcinoma, pheochromocytoma, and mucus fibroids. Total thyroidectomy for bicentric medullary thyroid carcinoma. Currently disease free. Primary hypothyroidism after thyroidectomy.  2019: reappearance of neurofibromas at the language level. Neurofibroma has also at ocular level. | 2016: Total thyroidectomy piece (10 gr; right lobe 3,5x1,5x1,2 cm; left lobe 2,8x1,5x1 cm).  Thyroid gland with multifocal and bilateral medullary carcinoma, exclusively intrathyroid, whose largest nodule is 0,5 cm. Intrathyroid thymus without alterations. Absence of metastasis in the 6 lymph nodes isolated from the central and cervical emptying. Buccal mucosa with 5 plexiform neurofibromas. | 2020: Discrete elevation of current calcitonin since 2016. | 2016: Neck ultrasound: two nodules in 5 and 7 mm lobes, no lymphadenopathy.  2020: Neck ultrasound: In the surgical bed of the thyroidectomy, a markedly hypoechogenic lesion is identified with numerous pinpoint foci suggestive of microlithiasis. The lesion measures 35x12x18mm (TR axis by AP axis by CC axis) and shows no clear vascularity. The findings are suggestive of tumor recurrence. Subcentimetric lymph nodes with preserved hilum are observed in the laterocervical chains. |
| 20 | Female | 77 | Medullary thyroid carcinoma (sporadic).esporadico T3N0M0 | Sporadic | No | Normal: Non neoplastic thyroid; Tumoral: 80-90% tumor + stroma | Family history: 1 brother died of pancreatic cancer 1 brother with liver cirrhosis Not know thyroid pathology Personal history: Primary hypothyroidism. | 2017: Thyroidectomy piece (right lobe measures 4,5x3,5 cm; left thyroid lobe 4x2,5 cm). When cut, a lesion (4,3 cm) is identified that occupies most of the right thyroid lobe, with homogeneous, brownish-whitish tissue.  Diagnosis: - medullary carcinoma of 4.3 cm. that sits on the right lobe, unifocal and intrathyroid. Vascular invasion. C-cell hyperplasia. Lymphocytic thyroiditis. Sixteen lymph nodes free of carcinoma infiltration. | Calcitonin more than 2000 before the intervention CEA more than 200 2018: Calcitonin with slight increase. 2020: Calcitonin has not gone up. | 2016 Thyroid ultrasound: Increases the size of the thyroid lobe at the expense of a single solid nodule of 2,7 cm with a slightly heterogeneous echogenicity. Small nodule in the left lobe of 3 mm of spongiform echogenicity. 2017 CT SCAN: Large heterogeneous hypercaptor right thyroid nodule (32 x 35 x 46 mm). No calcifications inside. Multiple lymph nodes of small size and non-pathological characteristics (both laterocervical chains).  At the lung parenchymal level, a 5 mm nodule at the level of the right lower lobe, with gross calcification inside, in relation to calcified granuloma. Degenerative changes at the level of C6-C7. 2020 Neck Ultrasound: Changes secondary to total thyroidectomy without evidence of persistence of glandular remains in the surgical site or appearance of new asymmetries or nodules suspected of nodular implants. |
| 21 | Female | 16 | Multifocal medullary thyroid carcinoma. TNM: T1N0M0 | MEN2A | C634Y | Normal: Tiroides no neoplásico T: 60-70% tumor + stroma + tiroides no neoplásico | Family history: Maternal family and mother MEN 2A syndrome. | 2007: Total thyroidectomy: Thyroid gland (10 gr) and that externally does not present relevant alterations. When serially cut, we can see a solid nodule with irregular edges measuring 5 mm. in the upper right pole. Diagnosis: bilateral and multifocal medullary carcinoma whose largest nodule is 1 cm (left lobe). the tumor is exclusively intrathyroid and the rest of the gland shows nodular C-cell hyperplasia. | Since intervention in 2007, calcitonin below 2 pg / ml. It has not gone up. Thyroid-stimulating hormone increased in 2018 and 2019. | No data available |
| 22 | Male | 26 | Medullary thyroid carcinoma. TNM: pT1a, pN1a, pMx | MEN2A | C634Y | Normal: Non neoplastic thyroid; Tumoral: 60-70% tumor + stroma + non neoplastic thryoid | 2012: Total thyroidectomy and central left and laterocervical lymph node emptying. Postsurgical dysphonia. Post-surgical hypocalcaemia. Paralysis of vocal chords. | 2012: Total thyroidectomy: The thyroid gland (right lobe 17,8 x 16,7 mm; left lobe 13 x 13,2 mm; thyroid isthmus 2,5 mm). - multicenter, located in both lobes, with the largest nodule of 0.7 cm in diameter. - Contact with thyroid capsule without exceeding it or reaching extrathyroid tissue. - rest of the thyroid parenchyma with foci of diffuse and nodular C-cell hyperplasia. - parathyroid glands without relevant alterations. - a perithyroid lymph node without relevant alterations. - resection margins free of neoplasia. - two thyroid tissue nodules without relevant alterations.  - thirteen lymph nodes, one of them with micrometastasis of medullary carcinoma, which does not exceed the capsule, and with a focus of angioinvasion in a small capsular lymphatic vessel. | 2012: Inmunohistochemical study: - Calcitonin: positive. - Thyroglobulin: negative.  2014: Thyroid hormone control to achieve normal TSH. | 2011 Neck ultrasound: Several nodular lesions are identified in both thyroid lobes. The largest of them is in the middle region and presents a mixed, cystic component with an eccentric solid area (11x8,6 mm). The remaining three nodules (7,3 mm, 5,2 mm and 6,8 mm). In the left thyroid lobe, we identified a single strong and hypoechogenic nodular lesion, markedly hypervascular and with poorly defined contours, with a blurred interface between the nodule and the healthy glandular parenchyma (8,8x 8,5 mm). These sonographic characteristics force to rule out malignancy. 2014: Neck: level 2 right cervical lymphadenopathy, small rolling soft tissue (<1 cm). |
| 23 | Female | 8 | Multifocal medullary thyroid carcinoma. TNM: Not available | MEN2A | C634Y | Normal: Non neoplastic thyroid; Tumoral: 70-80% tumor + stroma + 5% non neoplastic thyroid | Family history: MEN 2A Personal history: In 2000 Total thyroidectomy. Multicentric medullary carcinoma in both lobes, with focus larger than 2 mm and limited to the gland. No post-surgical complications.  2012: Hypothyroidism. 2005: Hyperplasia of adrenal glands. | Thyroid gland 4x2x1 cm. In the left thyroid lobe, a well-defined whitish nodule of 0,5 cm in diameter is identified. The serial cut in the right lobe identifies other nodules with similar characteristics to those described, which do not exceed 2 mm in diameter and are close to each other. | Surgically operated in 2000. During all these years: normal thyroid function and thyrocalcitonin levels below 2 pg / ml. | 2008 Medullary adrenal gammagraphy: High uptake by the liver parenchyma, salivary glands, myocardium and renal uptake. A slight increase in the uptake in both adrenals, suggestive of hyperplasia of the same. At the anterior and middle region of the neck, a focus of tracer uptake is evident, which probably corresponds to remains of thyroid tissue.  2009 Lateral Chest Radiography: within normal limits. |
| 24 | Female | 47 | Medullary thyroid microcarcinoma (sporadic), and a papillary thyroid cancer (TNM: T3N0M0) | Sporadic | No | Normal: Non neoplastic thyroid; Tumoral: 40-50% tumor + stroma + non neoplastic thyroid | Family history Not know thyroid disorders Personal history: - Steinert's myotonic dystrophy. - Hiatal hernia. - Diabetes Mellitus type II (2005) - Dyslipidemia without treatment. - Surgeries: Cholecystectomy, tonsillectomy and adenoidectomy. | Total thyroidectomy: Thyroid gland (38 gr; right lobe 5 x 3,5 cm; left lobe 4,5 x 2 cm).  Two simultaneous carcinomas, of different histological type, have been found in the same gland: 1 - 0,5 cm medullary carcinoma (right lobe) associated with foci of irregularly distributed C-cell hyperplasia. Unifocal and intrathyroid. A papillary carcinoma, of conventional histological type, multifocal. One of the nodules exceeds the glandular limits and minimally extends to perithyroid soft tissues. The cystic-hemorrhagic nodule (2,5 cm) is an adenoma of macrofollicular architecture. High-grade neoplasm showing p53 overexpression. It widely affects the right ovary and uterine tube.  2018: MLH1/PMS2 and MSH2/MSH6 nuclear expression in adenocarcinoma. Stage IIA of the FIGO. | This tumor is positive for calcitonin and CEA and is negative for thyroglobulin 2012: Slightly elevated thyroid stimulating hormone. Must maintain TSH levels braked, with normal free T4. | 2008: nodular goiter in right lobe. Euthyroidism 2012: Neck No glandular debris or nodules are identified in the thyroidectomy bed that suggest the existence of locoregional recurrence. No lymph node metastasis. Rest of cervical structures without alterations. |
| 25 | Female | 45 | Multifocal medullary thyroid carcinoma. TNM: T2N1M0 | MEN2A | C634Y | Normal: Non neoplastic thyroid; Tumoral: 70% tumor + stroma + non neoplastic thyroid | Family history: Healthy father and mother, both with hypertension. Father with symptomatic kidney stone disease. A sister with kidney stones. A brother with a kidney cyst. Rest healthy. No history of thyroid disease or early cardiovascular disease. Personal history: Congenital monorrena. Symptomatic renal lithiasis.  Posturgical hypoparathyroidism. | Adrenalectomy: 1 cm pheochromocytoma on the medulla with diffuse and nodular hyperplasia. Adrenal gland (13 mg; 6x4x0,7 cm). When serially cut, a well-defined nodule is observed, surrounded by an orange rim, a grayish color with hemorrhagic areas and measuring 1 cm.  2009: Total thyroidectomy piece (16 gr; both lobes same size). Multicenter and bilateral medullary carcinoma, exclusively intrathyroid, (largest nodule is 2 cm). - diffuse and nodular hyperplasia of C cells. - metastasis of medullary carcinoma in 2 of 3 central emptying nodes. 2019: Local recurrence of left pheochromocytoma in a patient with MEN2A syndrome with elevated metanephrine. | Calcitonin: positive in neoplasia and hyperplastic cells. Calcitonin in progressive decline. 2012: Very significant elevation of homovanilinic acid with elevated metanephrine and normetanephrine (upper limit). | Thyroid ultrasound: 1,4x1cm nodule in thyroid (left lobe), cystic. Another similar nodule in right lobe (0,5x0,4cm). PET / CT scan: No tracer deposits at the cervical level that could suggest locoregional recurrence or lymph node involvement. 2010 Thyroid Ultrasound: Non-pathological examination. 2010 Neck Ultrasound: There are three sub-centimeter nonspecific nodes with benign ultrasound criteria on both sides of the neck, two located on the right side and one on the left side. 2018: PET DOPA:  Left pheochromocytoma. |
| 26 | Female | 37 | Multifocal medullary thyroid carcinoma. TNM: T3N0M0 | MEN2A | C630Y | Normal: Stroma, blood vessels, non neoplastic thyroid; Tumoral: 90% tumor + stroma + non-neoplastic thyroid | Family history: Paternal cousin who has died suddenly at age 38y.  No family thyroid disease Personal history: Rheumatoid arthritis Iron deficiency anemia. Anemia of chronic processes. Myoma hysterectomy in 2012. | Total thyroidectomy piece (55 gr, 5 x 3 cm right thyroid lobe; 5 x 3,5 cm left one). Smooth external surface and full capsule. When the right lobe is cut, three brownish nodular formations are identified, homogeneous in appearance (1 cm the smallest and 3 x 3 cm the largest), poorly delimited. The left lobe is multinodular in appearance with nodules (0.5-3.8 cm), brownish coloration with some cystic cavities. Absence of extrathyroid extension. | 2012: Immunohistochemical study: calcitonin and CEA positive. negative thyroglobulin.  Clear decrease of Calcitonin after intervention. Upward trend in 2020. | 2012 Thyroid Ultrasound: A markedly enlarged thyroid, heterogeneous, with multiple nodules with a tendency to confluence, mainly isoechogenic, with cystic regions and echogenic pinpoint images with comet tail artifact by concentrated colloid. There are no significant cervical adenomegalies. Conclusion: Multinodular goiter. 2013 Lateral Chest Radiography: within normal limits. |
| 27 | Female | 67 | Unifocal medullary thyroid carcinoma (sporadic). TNM: T2N0M0 | Sporadic | No | Normal: Non neoplastic thyroid; Tumoral: 90% tumor + stroma + non neoplastic thyroid | Family history: No history of pituitary or thyroid pathology. Hypertensive father. Esophageal cancer mother. No catecholaminergic clinic. Personal history: Hypertension Tubular adenoma with low-grade dysplasia (2018). | 2012: Total thyroidectomy: Total thyroidectomy piece (25 gr. Right lobe 4 x 2,5 cm and left lobe 5 x 1,5 cm).  Exclusively intrathyroid medullary carcinoma measuring 3 cm in maximum size; one lymph node and a parathyroid without alteration. | Immunohistochemical study: - Calcitonin and CEA: Positive. - Thyroglobulin: Negative. | CT scan of the chest: 20 mm diameter solid-looking hypocritical nodule in right lobe.. 2011 Thyroid ultrasound: A subcentimetric nodular image is visualized in both thyroid lobes (1,6 x 1,4 x 2,4 cm). |
| 28 | Female | 60 | Medullary thyroid carcinoma with metastasis (sporadic). TNM: T3N1M1 | Sporadic | No | Normal: Non neoplastic thyroid; Tumoral: 80-90% tumor + stroma | Family history: Deceased father of ischemic heart disease Sister with papillary carcinoma Personal history: Hepatitis in childhood. Malta fever at 18y. Appendectomy. Hysterectomy. Total thyroidectomy in 2002 for medullary thyroid cancer. 2009: liver (segmentectomy VII and VIII): Binodal metastasis of medullary thyroid carcinoma with the presence of associated amyloid material that does not affect the soft tissue resection planes and lymph nodes. 2011: recurrence of pre and paratracheal soft tissue medullary carcinoma. Metastasis of medullary carcinoma in the five isolated nodes. Sporadic medullary thyroid carcinoma operated on 4 times. Post-surgical hypoparathyroidism. | No available data (surgery in 2002). | 2002: Calcitonin 1358.00 pg / ml 2009: Calcitonin: Expression in carcinoma cells.  2011: Persists with disease (elevated Calcitonin) not localized 2017: Progressive elevation of calcitonin, although slowly. | 2016 Neck ultrasound: A homogeneous hypoechoic solid nodule (17 x 9 mm). In a right lateral situation to this: two other nodules of similar appearance are observed (the largest 12,5 x 9,5 mm; the smallest 6,5 mm), with a scarce central vascularization, findings that suggest the presence of metastatic lymphadenopathy in visceral space.  2017 Neck ultrasound: no suspicious nodes in right lateral cervical chains. In the left laterocervical region (spaces III and IV), lymph nodes are observed without evidence of a clear hilar vascularization pattern. These findings in the current clinical context of the patient raise the suspicion of possible metastatic involvement. PET with F Dopa:  Doubtful hypermetabolic injury in liver segment VIII. Rest of the study without pathological findings. |
| 29 | Female | 38 | Multifocal medullary thyroid carcinoma. TNM: T3N1aM0 | MEN2A | C634Y | Normal: Non neoplastic thyroid; Tumoral: 70-80% tumor + stroma + non neoplastic thyroid | Personal history: Medullary Thyroid Carcinoma with elevated calcitonin (total thyroidectomy). Bilateral Pheochromocytoma (2-stage bilateral adrenalectomy). Patient undergoing endocrine follow-up and presented to a committee of tumors for MEN 2A syndrome undergoing total thyroidectomy in 2005: without lymph node emptying due to medullary thyroid and bilateral adrenalectomy due to pheochromocytoma, presenting increasing calcitonin. | No thyroid tumor data 2009: Pheochromocytoma in a patient with MEN IIA syndrome: Adrenal gland (17 gr) with a nodule (1,4 cm) that, when cut, is in the medullary portion, well delimited, surrounded by the cortical orange border, is dark yellow, with black areas and elastic consistency. Adrenalectomy: pheochromocytoma, without signs of histological malignancy, measuring 1,4 cm. Spinal hyperplasia. Cortical hyperplastic adenoma-nodule (0,3 cm).  2018: Lymph node emptying: 4/4 lymph nodes with metastasis from medullary carcinoma. (the largest measures 0,7cm, with extranodal extension). | Immunohistochemical study, with a positive result for calcitonin and TTF1 in carcinoma, which is consistent with the diagnosis. Persistence with elevated calcitonin (low after intervention but then rise again). | 2017 PET/ CT SCAN: two pathological foci of tracer hyper-uptake were found, in the left thyroid bed (5 - 6 mm). These lesions are suggestive of malignancy. Moderate uptake in striated bodies and pancreas, and accumulation in ureters, bladder and gallbladder due to physiological elimination of FDOPA, without other foci being detected. |
| 30 | Female | 50 | Medullary thyroid microcarcinoma and papillary thyroid carcinoma (TNM: T2N0M0) | Sporadic | No | Normal: Tiroides no neoplásico T: 40% tumor + stroma + tiroides no neoplásico | Family history: No family history of thyroid disease. Father dead with lung cancer.  Personal history:  Surgery of papillary thyroid carcinoma (2003), without disease data (Low Risk, excellent response) Microscopic medullary microcarcinoma. Current status: disease free. | 2003: right hemithyroidectomy for nodular goiter. A 2,3 cm thyroid papillary neoplasm that infiltrates the capsule without exceeding it and does not affect the resection border.  Left hemithyroidectomy. No post-surgical complications. | Thyroglobulin and undetectable calcitonin in hypothyroidism. | 2005 Scan with 131I: Foci of pathological uptake that suggest the presence of local or metastatic disease. Only the activity that corresponds to the physiological elimination of the tracer is observed, urinary via the kidney or digestively via the salivary glands, mainly.  2007 and 2008 Neck Ultrasounds: no findings. 2011 Neck ultrasound: Small hypoechoic nodule in the right visceral space. Nodes of size and non-pathological appearance homogeneously distributed by cervical spaces, predominantly submaxillary and in internal jugular chains.  2012 Neck Ultrasound: A small well-defined hypoechoic and homogeneous 4,5 mm nodule persists in the right paratracheal situation. At the different lymph node levels, there are nodes (3,5 and 7 mm), most with visible fatty hilum, without pathological characteristics. |
| 31 | Male | 19 | Multifocal medullary thyroid carcinoma. TNM: Not available | MEN2A | C634Y | Normal: Non neoplastic thyroid; Tumoral: 35% tumor + stroma + non neoplastic thyroid | Family history: Mother and maternal uncles with MEN 2A syndrome Personal history: Hypothyroidism Total thyroidectomy, no disease data 2012: Right adrenal pheochromocytoma (right adrenalectomy) 2019: Left adrenalectomy with retroperitoneal approach.  No postsurgical complications. | Resection piece of the right adrenal (30 gr). The tumor has a brownish coloration, a friable consistency and is 5x3 cm. The tumor is surrounded by a thin fibrous capsule and appears to depend on the adrenal gland, with no other significant gross abnormalities.  2006: Total thyroidectomy piece (15 gr) and in which there is a larger lobe and a smaller one. The external surface is smooth and there is no evidence of rupture of the thyroid capsule. Diagnosis: thyroid gland (total thyroidectomy): - bilateral multi-central spinal carcinoma, intrathyroid, whose major focus measures 0,7 cm. - nodular and diffuse C cell hyperplasia. | Calcitonin: Positive in neoplastic and hyperplastic C cells. | MIBG scan:  A 20x15 mm node dependent on the arm lateral of the left adrenal gland. These findings are suggestive of pheochromocytoma in the left adrenal gland. Left adrenal hypermetabolic lesion consistent with suspected left pheochromocytoma. Rest of the study without pathological findings. |
